# Rare malignant primary spinal chondrosarcoma: A case report

**DOI:** 10.1016/j.radcr.2024.10.127

**Published:** 2024-11-22

**Authors:** Ahmad Fitrah, Btari Magistra Pancaputri, Andreas Klemens Wienanda, Atta Kuntara, Abdul Kadir Hadar, Muhammad Naseh Sajadi Budi Irawan, Ahmad Ramdan, Anglita Yantisetiasti

**Affiliations:** a Department of Radiology, Faculty of Medicine Padjadjaran University. Dr. Hasan Sadikin Hospital, Bandung, West Java, Indonesia; b Department of Orthopedic, Faculty of Medicine Padjadjaran University. Dr. Hasan Sadikin Hospital, Bandung, West Java, Indonesia; c Department of Pathology Anatomy, Faculty of Medicine PADJADJARAN University. Dr. Hasan Sadikin Hospital, Bandung, West Java, Indonesia

**Keywords:** Chondrosarcoma, Case report, Spine

## Abstract

Chondrosarcomas are one of malignant tumors in which cartilaginous matrix is produced. It is divided into 2 groups including primary or secondary. Primary chondrosarcomas are the third most common primary malignant tumors of the bone. Chondrosarcoma represents 20%–27% of all primary malignant bone tumors. Primary spinal chondrosarcoma is exceedingly rare among spinal tumors. A 36-year-old man presented to hospital with the swelling on the back accompanied with pain. Swelling and pain have been felt for approximately 3 years. The symptoms gradually worsened. On thoracolumbal X-ray a lytic sclerotic expansile lesion on the right posterior aspect of thoracal T5-T6. MRI showed the mass infiltrated intradural and intramedullary, reaching up to the level of vertebrae T4-T5. This infiltration resulted in stenosis of the spinal canal, obliterating the ligamentum flavum, supraspinous ligament, and interspinous ligament. Chondrosarcomas are uncommon malignant bone tumours that form cartilage; they rarely involve the spine, while most of them occur in young men. The thoracic spine is most commonly involved, but there is usually a long history of pain and possible neurological symptoms. Imaging techniques, such as conventional examination, CT, and MRI, are very important for diagnosis and classification and show typical bone destruction with matrix mineralization. Imaging revealed a lytic sclerotic lesion at the T5-T6 level. CT scans performed subsequently showed an expansile mass with a typical ``rings and arcs'' appearance of chondrosarcomas. MRI further delineated the extent of the mass and the surrounding tissue infiltration, and confirmation of low-grade chondrosarcoma, grade I was based on histological examination. The most effective treatment has been en bloc resection, and high-dose adjuvant radiotherapy might improve local control and survival rates. Recommended follow-ups are for the purpose of monitoring recurrence. Primary spinal chondrosarcoma is a rare malignant tumor that predominantly affects adolescents. The standard treatment typically involves surgical intervention, often supplemented with adjuvant radiotherapy. Many patients experience considerable improvements in neurological function following treatment. Long-term monitoring and follow-up are crucial for ensuring the best possible outcomes for individuals with primary spinal chondrosarcoma.

## Case presentation

A 36-year-old man presented to hospital with the swelling on the back accompanied with pain. Swelling and pain have been felt for approximately 3 years. The symptoms gradually worsened; he felt numbness and weakness of both lower limbs with hypoesthesia, constipation, difficult to squat, and the swelling was increasing in size. The neurological examination revealed normal findings with grade 4/5 of bilateral lower limb strength, and hypesthesia below the xiphoid level. The pathological reflex examination revealed positive Babinski and Romberg signs. The previous surgical or trauma history and family history findings were not significant.

**Fig. 1:**
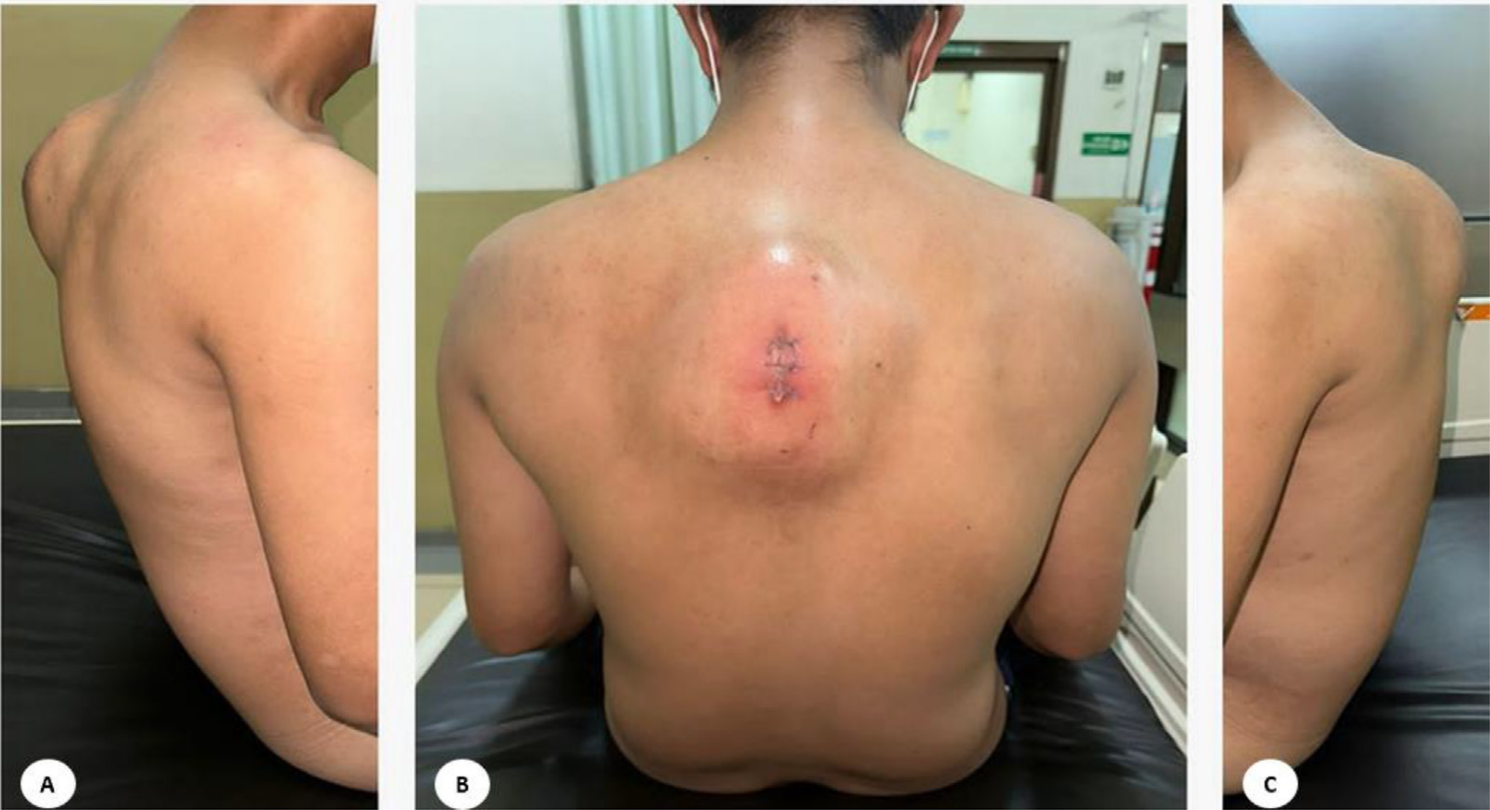
Clinical pictures of a 36-year-old man with complaints of swelling and pain. (A) Posterior right lateral view, (B) Posterior view lesion with post biopsy scar, (C) Left lateral view.

The physical examination of posterior chest revealed deformity, gibbous, lump and post biopsy scar. The patient underwent an incisional biopsy at a hospital 2 months prior. After the biopsy, the patient complained of an enlarging lump and pain, leading to a referral to our hospital. Tenderness along the midline was detected, while both sensation and movement tests returned normal results. The neurological examination also revealed hypoesthesia at the T4 level (Fig. 1).

**Fig. 2:**
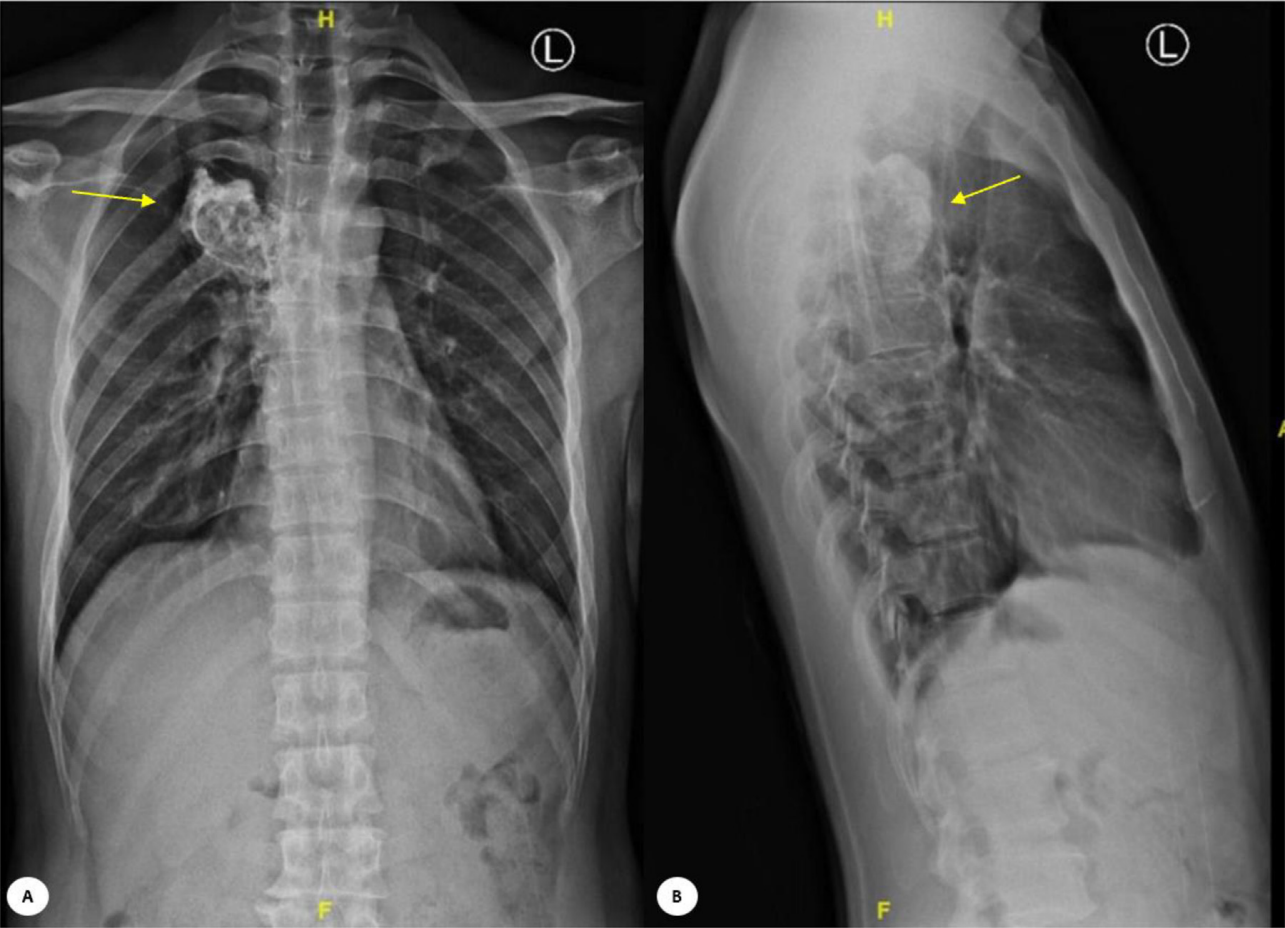
Conventional x-ray examination of the thoracolumbar spine (X- ray Carestream DRX Ascend 2018 with (A) Anterior-posterior and (B) Lateral view revealed a lytic sclerotic expansile lesion on the right posterolateral aspect of the thoracic vertebrae 5-6 (yellow arrow).

In the conventional X-ray examination (CR) of the thoracolumbar spine, anterior-posterior and lateral views revealed a lytic sclerotic expansile lesion on the right posterior aspect of thoracal T5-T6 (Fig. 2). The curvature and alignment of the thoracolumbar bones were within normal limits. The size, shape, and trabecular structure of other thoracolumbar vertebrae were also normal.

**Fig. 3:**
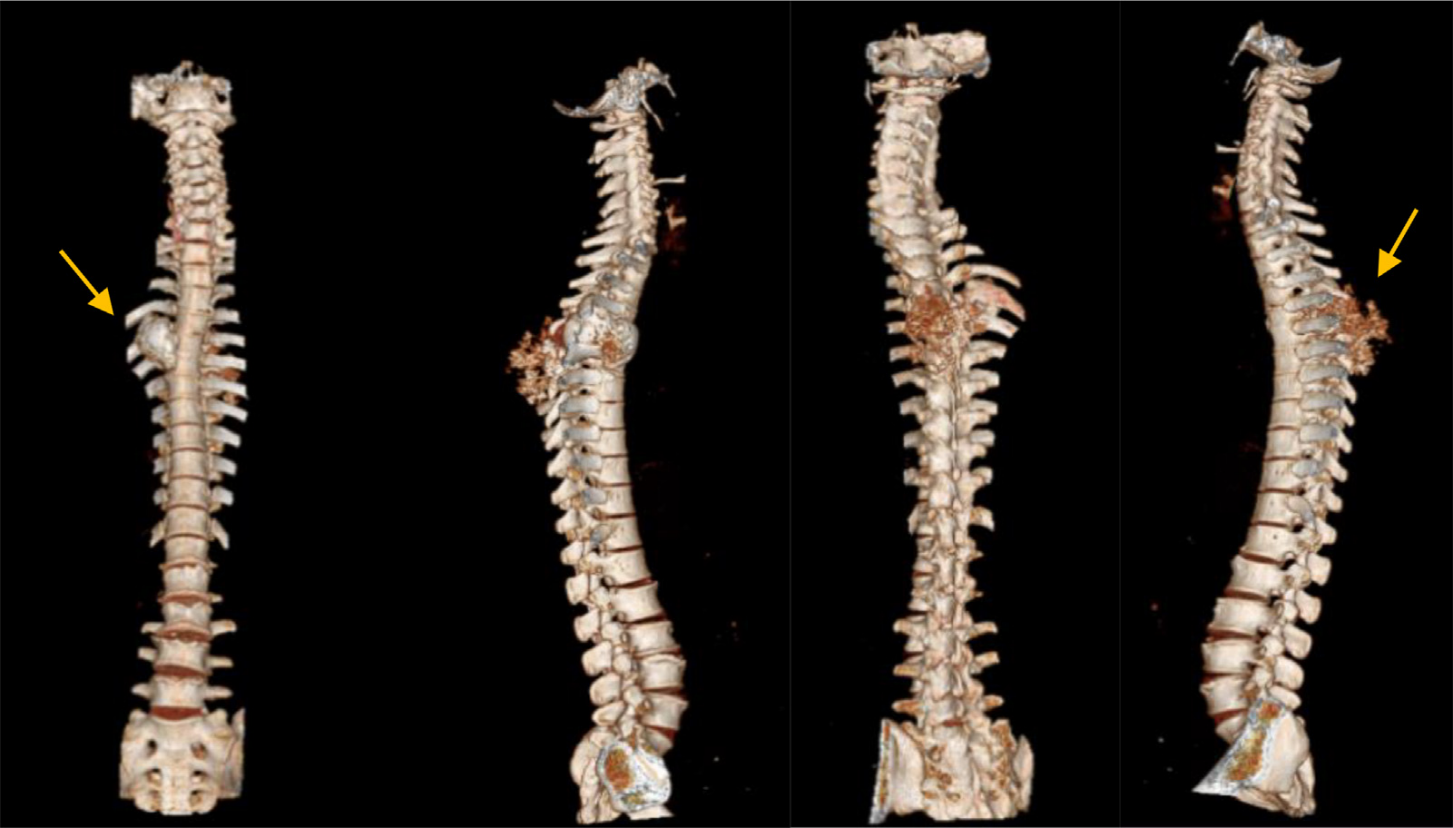
Computerized tomography scan of column vertebrae region with 3D construction (CT- Scan Hitachi Scenaria 2017). A mass located in the right posterolateral aspect of the thoracic vertebrae T5-T6 (yellow arrow).

**Fig. 4:**
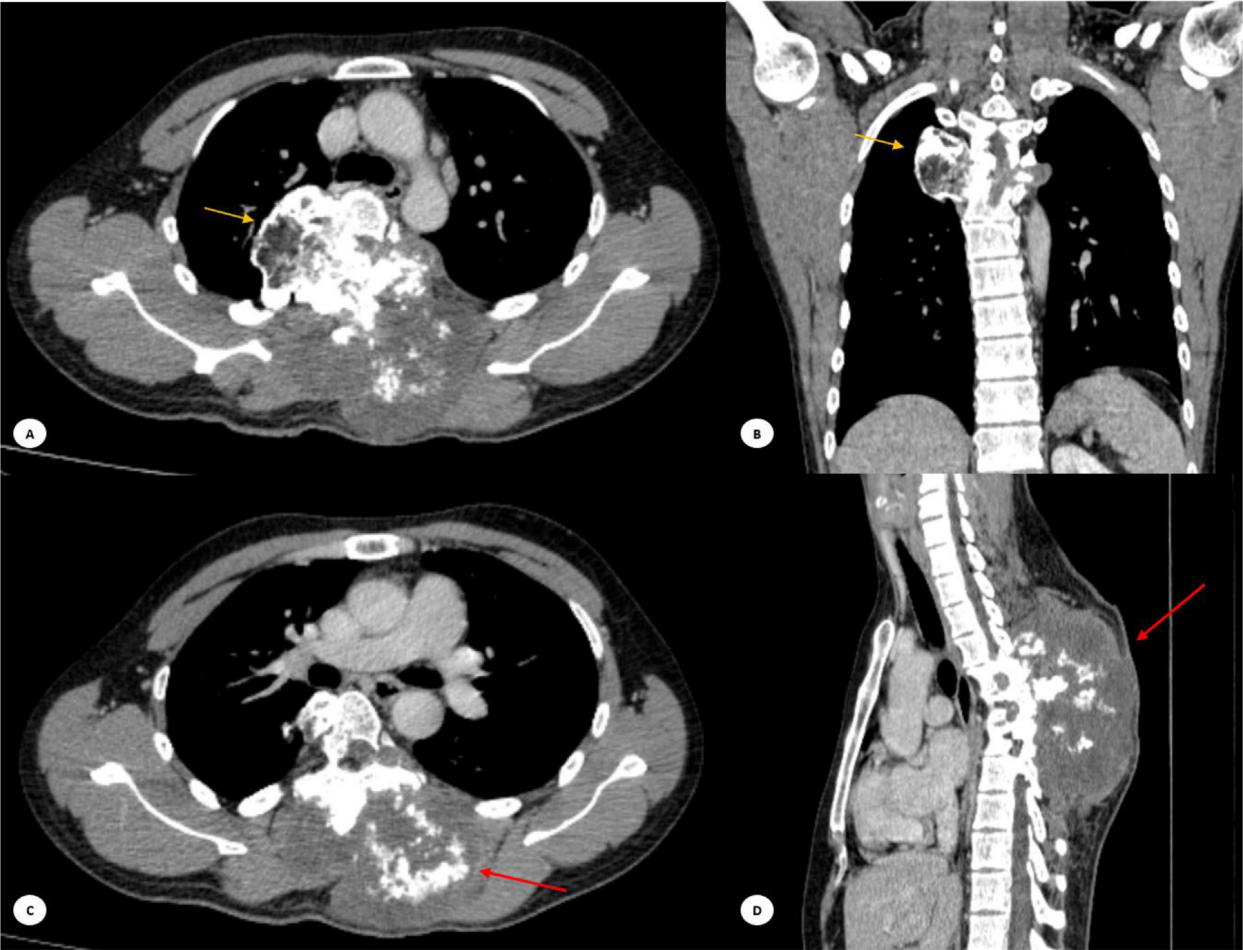

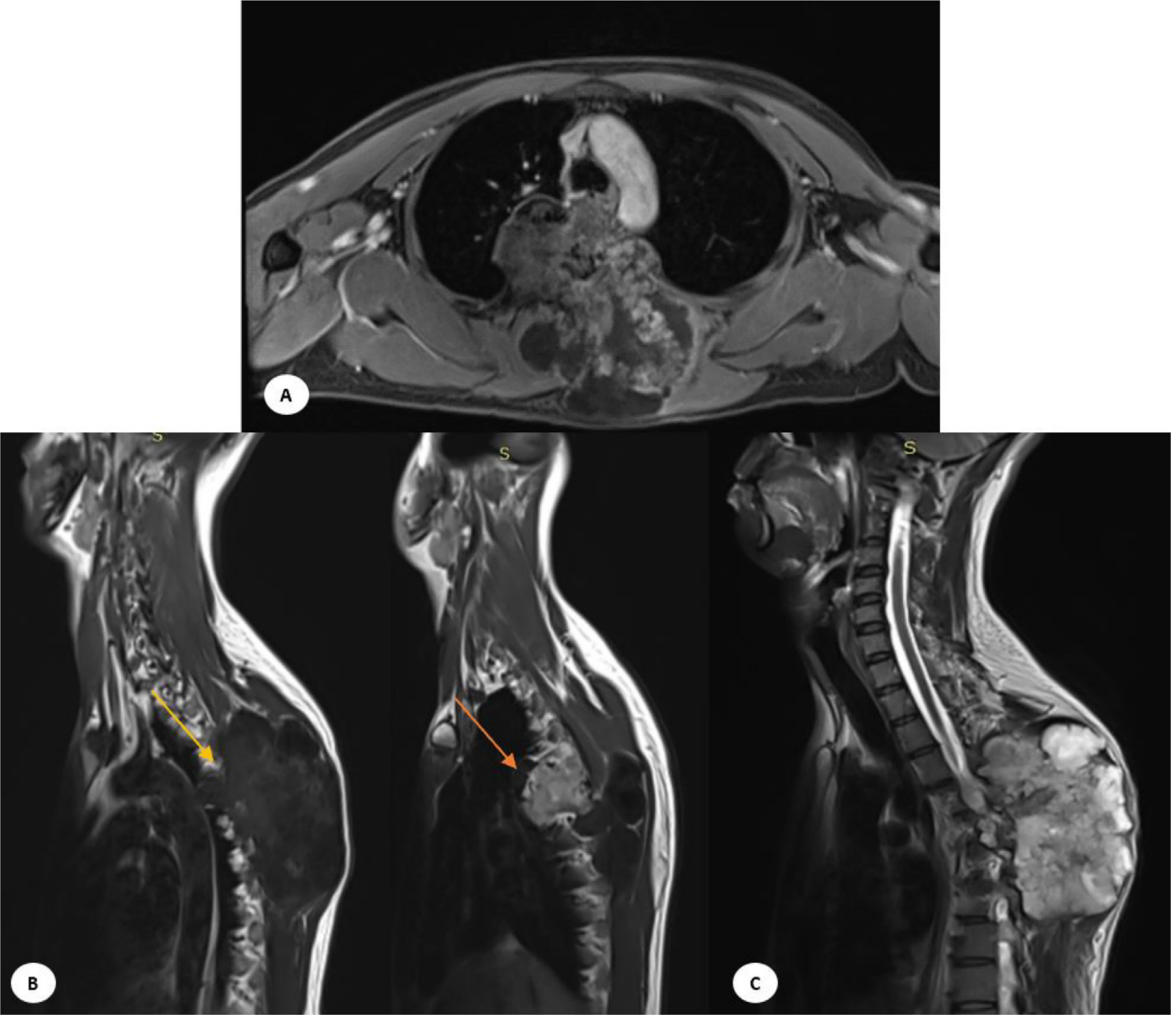
CT image in soft-tissue window (A, B) Axial and coronal view revealed in coronal and axial view the mass extending to the transverse process, costovertebral junction, and right posterolateral aspect of the thoracic vertebrae 5-6 (yellow arrow). (C, D) Axial and sagital view revealed an expansile sclerotic mass with a chondroid matrix located in the right posterolateral aspect of the thoracic vertebrae 5-6 (red arrow). MRI of the thoracolumbar region (MRI 1.5 T siemens Magnetom Essenza 2012). (A) T1-weighted imaging showed low signal intensity mass extending into the subcutaneous tissue in the bilateral posterior region of the vertebrae on axial image, (B) The mass infiltrated intradurally and intramedullary, reaching up to the level of vertebrae T4-T5, also stenosis of the spinal canal on sagittal image, on the left side, it directly bordered the aortic arch (yellow arrow) and the mass extended into the thoracic cavity (orange arrow), (C) T2-weighted showed high signal intensity partial mass with enhanced septations after intravenous injection of contrast media.

The patient underwent a computerized tomography (CT) scan of the spine with axial, coronal, and sagittal views, using a 1.3 mm slice interval. The scanning was performed both with and without contrast, and a 3D reconstruction was created. The CT scan revealed an expansile sclerotic mass with a chondroid matrix located in the right posterolateral aspect of the thoracic vertebrae T5-T6, extending to the transverse process, costovertebral junction, and posterior costae 5-6. The dimensions of the mass were 4.85 × 5.03 × 11.87 cm, accompanied by soft tissue swelling and calcification in the surrounding area (Fig. 3, Fig. 4).

Other trabecular structures in the thoracolumbar bone were within normal limits. The intervertebral discs in other scanned thoracic spine areas were also normal. The shape and structure of the pedicles appeared normal. No osteophytes were observed on the endplates of the vertebral bodies. The spinal cord (medulla spinalis) and other components of the spinal canal were within normal limits (Fig. 3).

Based on the CT scan findings, the diagnosis is suggestive of a primary bone tumor, with differential diagnoses are chondrosarcoma, osteosarcoma and osteochondroma.

The patient also underwent magnetic resonance imaging (MRI) of the thoracolumbar region with axial-sagittal T1-weighted imaging (TIWI), sagittal T2-weighted Dixon Water, T2-weighted Dixon Fat, T1-weighted Dixon Water, and T1-weighted Dixon Fat sequences. The scanning was performed both with and without contrast. During this examination, an inhomogeneous mass was identified. It had irregular sharp margins and contained necrotic tissue. The mass measured approximately 12.99 × 7.63 × 13.10 cm and appeared to originate from the bone. Specifically, it involved the vertebrae T5-T6, extending into the subcutaneous tissue in the bilateral posterior region of the vertebrae, spanning from T4-T8. The mass caused destruction of the spinous processes, transverse processes of the vertebrae, costovertebral junction, and posterior costae 5-6 (Fig. 4).

Furthermore, the mass infiltrated intradural and intramedullary, reaching up to the level of vertebrae T4-T5. This infiltration resulted in stenosis of the spinal canal, obliterating the ligamentum flavum, supraspinous ligament, and interspinous ligament. The mass also obliterated the bilateral m. spinal thoracis, m. iliocostalis thoracis, m. iliocostalis lumborum, m. rotators, and m. semispinalis thoracis. Anteriorly on the right side, the mass extended into the thoracic cavity, while on the left side, it directly bordered the aortic arch.

The curve and alignment of the thoracolumbar vertebrae appear to be normal. The corpus of other thoracolumbar vertebrae also maintains a normal shape and signal intensity. The intervertebral discs and articular facets of the other thoracolumbar vertebrae are within normal limits. The signal intensity of the nucleus pulposus and annulus fibrosus components of other intervertebral discs remains normal. Additionally, there are no pathological changes in the facet joints. These findings are consistent with chondrosarcoma (Fig. 4).

The surgery with a median posterior approach was conducted. A solid red mass was found in the right side of extramedullary thoracic region with the spinal cord destruction. When the arachnoid was opened, a stiff well-demarcated tumor that adhered weakly to the right side of thoracic was revealed. The en bloc tumor resection was performed including the removal of attached duramater and continued with histologic examination.

**Fig. 5:**
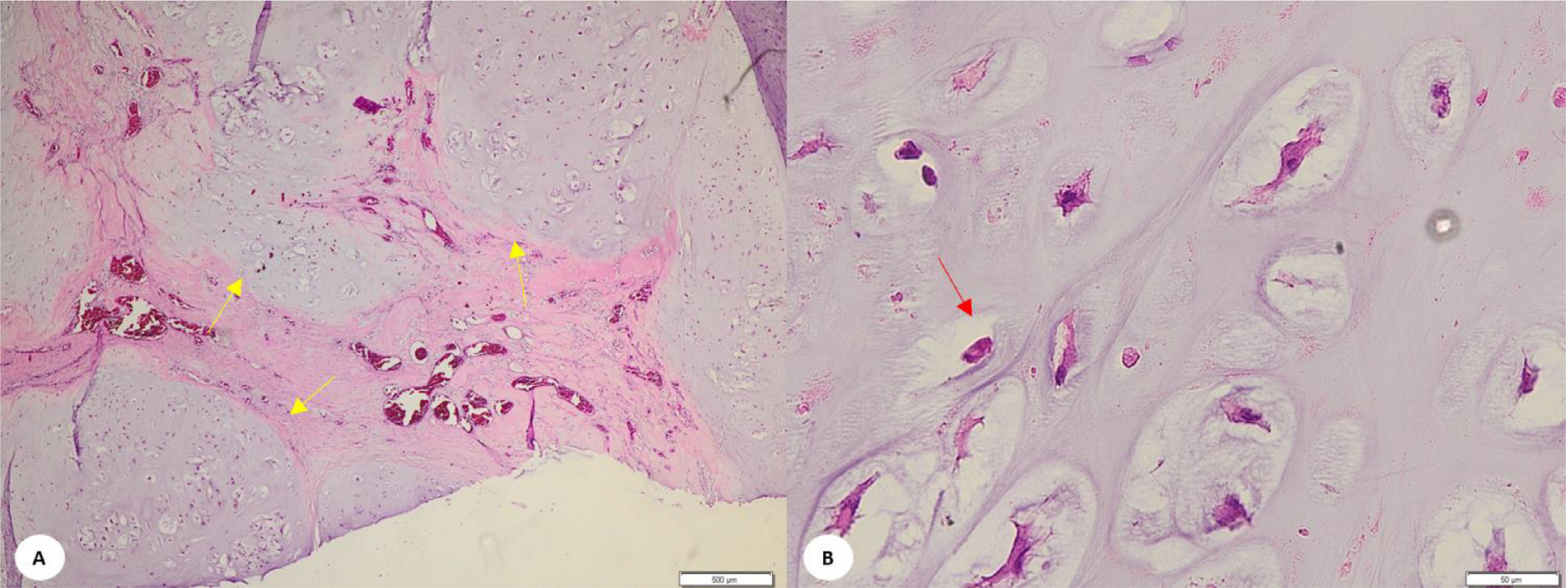
Histopathological examination of chondrosarcoma grade I. (A) Lobulated hyaline cartilage neoplasm (yellow head arrow), magnification H&E 20x, (B) Moderately cellular and contain hyperchromatic, pale, plump nuclei of uniform size (red arrow), magnification H&E 200x.

Histopathological examination revealed that the T4-T5 thoracic vertebral tissue were partially covered with keratinized stratified thin epithelium, and the nuclei were within normal limits. Subepithelial, there were tumor masses consisted of hyperplastic atypical oval-shaped cells. The nuclei of atypical cells were pale, plump and hyperchromatic, and mitosis was difficult to find. Among the tumor cells, a chondromyxoid cartilage matrix was observed. The tumor mass ware lobulated and separated by bone lamellae containing normal osteocytes. Additionally, osteoblastic rimming was present. Hematopoietic tissue (marrow) appeared between the lobes (Fig. 5).

The surgical incision edges showed a thin keratinized squamous epithelium, within normal limits. The nuclei subepithelial region consisted of stroma, connective tissue, fibro-collagen, and inflammatory cells (lymphocytes), along with blood vessel dilation and bleeding. Importantly, there was no visible infiltration of malignant tumor as mentioned above. The final diagnosis was chondrosarcoma grade I involving vertebrae thoracal T4-T6 in the thoracic spinal region, and the incision edges from the operation were free of malignant tumor cells.

**Fig. 6:**
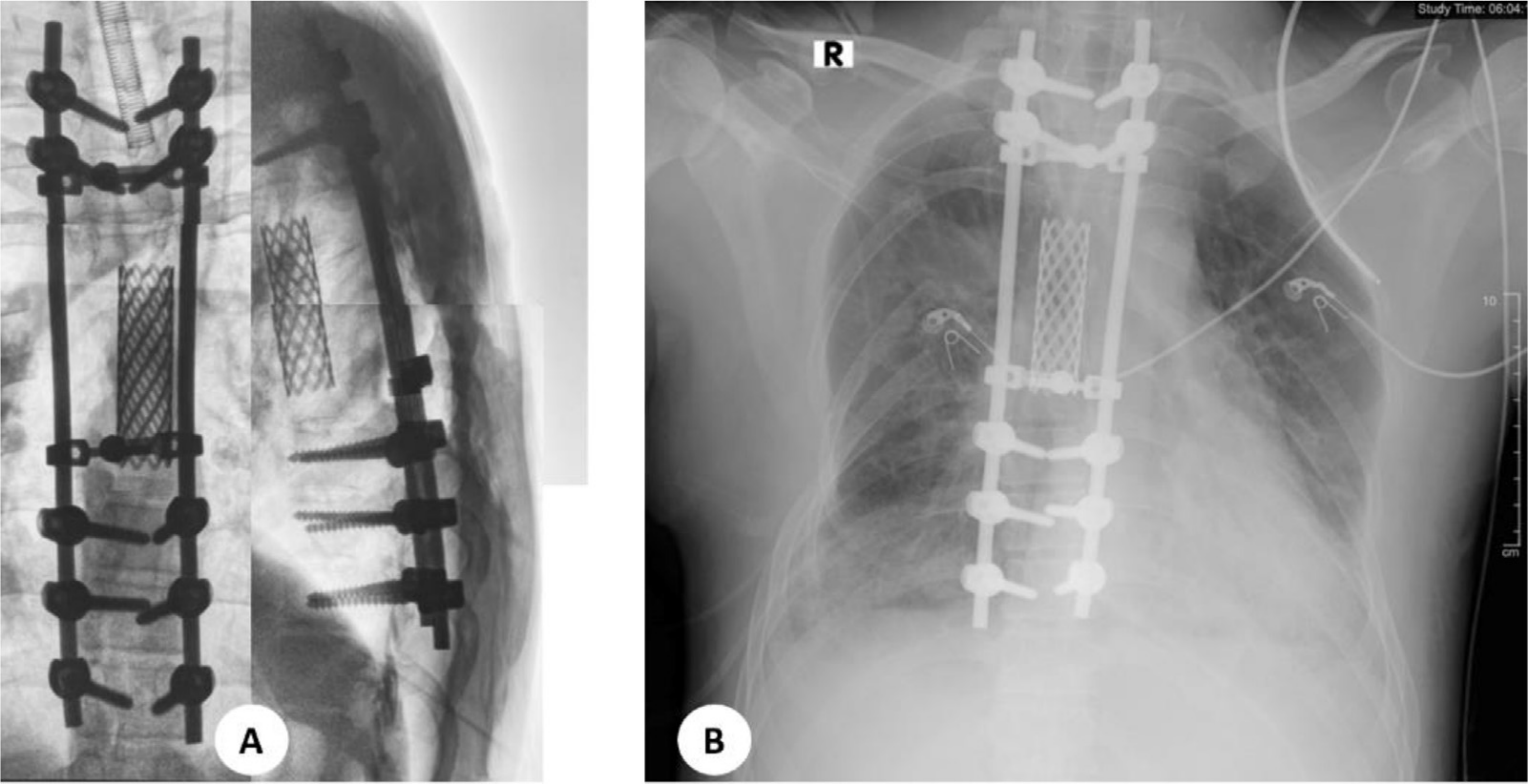
Postsurgery (A) Thoracolumbal X-ray imaging, the T5-T6 thoracic vertebrae bodies are not visualized due to corpectomy. (B) Thorax X-ray imaging, showed no complication from surgery.

Postoperative conventional radiographic examination of the patient's thoracolumbar spine and monthly follow-up is carried out to assess the patient's clinical condition. On conventional examination, the thoracolumbar radiograph showed deformity in the right posterior aspect of costae 4, 5, 6, and the left posterolateral aspect of the thoracic vertebrae T5-T7 after osteotomy. The T5-T6 thoracic vertebrae bodies are not visualized due to corpectomy. The patient was placed in posterior stabilization at the level of thoracic vertebrae bodies T2-T10. The mesh cage is visualized at the level of thoracic vertebrae T4-T7. There are no narrowing discs and other intervertebral foramina. The pedicles of the other vertebral bodies were within normal limits (Fig. 6A). The thoracic x-ray reveals no signs of pneumothorax, pneumomediastinum, or any postoperative complications (Fig. 6B). The patient's clinical condition and vital signs are good and stable, with minimal bleeding.

## Discussion

Chondrosarcomas are cartilage-forming bone neoplasm, these neoplasms rarely affected the spine. Primary spinal chondrosarcoma affects most frequently occurs during the second decade of life. Men are affected 2 to 4 times more frequently than women [[1], [2], [3], [4], [5], [6], [7], [8], [9], [10], [11]]. The thoracic spine is the most frequent localization (accounting for 60%), followed by the cervical and lumbar spine [4,[12], [13], [14]]. Chondrosarcomas are generally slow growing, patient often came with long term pain (for 1–2 years) and tenderness with or without a mass. Some patient also complaint a neurologic symptom [[2], [3], [6], [10], [13], [15]]. This case is similar with a case report by Wang et al, both patients had paraparesis with hypesthesia below the xiphoid level, Babinski sign, and Romberg sign without any complaint of palpable mass. The MRI examination revealed a small intradural mass at the dorsal side of 5^th^ thoracic (T5) canalis spinalis [5].

The characteristic imaging features of various chondrosarcoma types are essential for accurate diagnosis and classification, as evidenced by conventional x-ray examinations, computed tomography (CT) scans, and magnetic resonance imaging (MRI). Plain x-rays typically reveal bone destruction accompanied by distinctive chondroid matrix mineralization. CT imaging enhances evaluation of bone involvement, while MRI is critical for delineating soft tissue and intraosseous components of the tumor [5,6,12,14].

Conventional x-ray examination of chondrosarcomas revealed a predominantly lytic lesion with or without matrix mineralization and bone destruction. Spinal chondrosarcoma may present as a lytic lesion involving the vertebral body or compression fracture of the superior or inferior end-plates [[5], [6], [7], [12],^14^]. In our case, imaging revealed a lytic sclerotic expansile lesion on the right posterolateral aspect of the thoracic vertebrae T5-T6, though the lesion was obscured in the vertebral body due to overlying structures. Further evaluation with a CT scan demonstrated an expansile sclerotic mass with a chondroid matrix, located in the right posterolateral region of T5-T6, extending to the right transverse process, costovertebral junction, and posterior costae 5 and 6. This mass was associated with soft tissue swelling and calcification in the surrounding area, displaying the characteristic “rings and arcs” pattern typical of chondrosarcomas, which arises from mineralized chondroid matrix. Additionally, extension of the tumor through the disk was noted in approximately 35% of cases, potentially impacting adjacent ribs [6,7].

CT scans of chondrosarcomas typically show bone destruction, small calcifications, and the extent of the tumor both inside and outside the bone. CT is better than MRI at outlining bone structures and showing mineralized areas. Calcification patterns differ between low-grade and high-grade chondrosarcomas: low-grade tumors often have dense, ring-like or speckled calcifications, while high-grade tumors usually show more irregular, amorphous calcifications. An MRI later revealed an irregular mass with sharp edges and necrotic tissue originating from the T5-T6 vertebrae. This mass extended into the surrounding soft tissue, affecting areas from T4-T8, and caused damage to the spinous and transverse processes, as well as the costovertebral junction and posterior ribs at T5-T6. The mass also infiltrated the intradural and intramedullary spaces, consistent with primary chondrosarcoma. About 15% of chondrosarcomas occur in the vertebral bodies, and around 35% extend through the intervertebral disc to nearby vertebrae. This is supported by findings from Ollivier et al., which show that MRI effectively reveals medullary involvement. The MRI showed a lobulated lesion with low to intermediate signal intensity on T1-weighted images and high signal intensity on T2-weighted images. Low-grade lesions displayed a lobulated pattern with enhanced septations after contrast injection, while high-grade lesions lacked septations and showed more diffuse enhancement. MRI is especially useful for assessing bone marrow edema, soft tissue involvement, and spinal canal encroachment [[6], [7], [12]].

The mass at the T4-T5 vertebrae infiltrates both the intradural and intramedullary spaces, causing spinal canal stenosis and obliterating the ligamentum flavum, supraspinous ligament, and interspinous ligament. A study by Lloret et al. also notes tumor extension into the spinal canal. MRI is superior to CT in depicting epidural and intraforaminal extension, highlighting potential compression of neural structures. If not treated adequately, chondrosarcoma is likely to recur. The most effective treatment, associated with the lowest recurrence rates, is en bloc resection. In our case, we performed an en bloc tumor resection, including removal of the attached dura mater, followed by histologic examination [7,12,16].

The histologic grading of chondrosarcoma was classified based on the nuclear size, nuclear hyperchomasia, mitotic activity, and cellularity degree. The surgical margins and surrounding trabeculae also evaluated whether it is invaded by any chondroid tissue invading the trabecular bone. In our case subepithelial visible tumor masses consisted of oval-shaped cells that exhibited hyperplastic growth. The nuclei of atypical cells were pale, plump, and hyperchromatic, and mitosis was difficult to find. Among the tumor cells, a chondromyxoid cartilage matrix was observed. The final diagnosis was chondrosarcoma grade I, according to 2020 WHO classification of chondrosarcomas, a low-grade tumor containing chondrocytes with small dense nuclei. The stroma is predominantly chondroid with sparse or absent myxoid areas. Nonmineralized regions have a translucent appearance, reflecting the high-water content of hyaline cartilage. In clinical practice, grade 1 chondrosarcoma is the most commonly observed [2,14,[15], [17], [18], [19], [20]].

Using high-dose adjuvant radiotherapy can improve local control pain and improve neurological deficits. Furthermore, high dose adjuvant radiotherapy can also improve recurrence-free survival rates in high grade lesions. It is recommended to conduct follow-up examinations, including physical assessments and posterior-anterior/lateral CR or CT scans of the thoracic region, every 3–6 months for the first 5 years, followed by annual assessments for a minimum of 10 years [12,16].

## Conclusion

Primary spinal chondrosarcoma is a rare malignant tumor that predominantly affects adolescents. The standard treatment typically involves surgical intervention, often supplemented with adjuvant radiotherapy. Many patients experience considerable improvements in neurological function following treatment. Long-term monitoring and follow-up are crucial for ensuring the best possible outcomes for individuals with primary spinal chondrosarcoma.

## Patient consent

Written informed consent for publication of their case was obtained from our patient's.
